# Immobilization of Ni_3_Co Nanoparticles into N‐Doped Carbon Nanotube/Nanofiber Integrated Hierarchically Branched Architectures toward Efficient Overall Water Splitting

**DOI:** 10.1002/advs.201902371

**Published:** 2019-12-01

**Authors:** Tongfei Li, Sulin Li, Qianyu Liu, Jingwen Yin, Dongmei Sun, Mingyi Zhang, Lin Xu, Yawen Tang, Yiwei Zhang

**Affiliations:** ^1^ Jiangsu Key Laboratory of New Power Batteries Jiangsu Collaborative Innovation Centre of Biomedical Functional Materials School of Chemistry and Materials Science Nanjing Normal University Nanjing 210023 China; ^2^ Jiangsu Optoelectronic Functional Materials and Engineering Laboratory School of Chemistry and Chemical Engineering Southeast University Nanjing 211189 China; ^3^ Key Laboratory for Photonic and Electronic Bandgap Materials Ministry of Education School of Physics and Electronic Engineering Harbin Normal University Harbin 150025 P. R. China

**Keywords:** bifunctional electrocatalysts, electrospinning, hierarchical architectures, overall water splitting, transition metal alloys

## Abstract

Exploring cost‐effective and high‐performance bifunctional electrocatalysts for both hydrogen evolution reaction (HER) and oxygen evolution reaction (OER) is of paramount importance for the advancement of H_2_ production technology, yet remains a huge challenge. Herein, a simple electrospinning–pyrolysis strategy is developed to directly immobilize uniform Ni_3_Co nanoparticles into a hierarchical branched architecture constructed by in situ formed N‐doped carbon‐nanotube‐grafted carbon nanofibers. The elaborate construction of such hybrid hierarchical architecture can effectively modulate the electronic structure of the active sites, enlarge the exposure of active sites, and facilitate the electron transfer and mass diffusion, favoring both the HER and OER. As a result, the optimized catalyst requires relatively low overpotentials of 114 and 243 mV for HER and OER, respectively, to deliver a current density of 10 mA cm^−2^ in 0.1 m KOH electrolyte. When employed as a bifunctional catalyst for overall water splitting, the resultant catalyst shows a low cell voltage of 1.57 V to achieve a current density of 10 mA cm^−2^, along with an impressive stability without noticeable attenuation even after 27 h. This work presents a successful demonstration in optimizing the electrocatalytic performance of Ni‐based bifunctional electrocatalysts by simultaneously considering modulation of electronic structure, hybridization with carbon substrate, and nanostructuring through a facile synthetic strategy, which provides a new avenue to the design of a rich variety of robust transition‐metal‐based electrocatalysts for large‐scale water electrolysis.

## Graphical abstract

A feasible electrospinning–pyrolysis strategy is proposed to directly immobilize uniform Ni_3_Co nanoparticles into a hierarchical branched architecture constructed by in situ formed N‐doped carbon‐nanotube‐grafted carbon nanofibers (Ni_1.5_Co_0.5_ @ N‐C NT/NFs). Synthesized Ni_1.5_Co_0.5_ @ N‐C NT/NFs demonstrate excellent activity and remarkable stability as a bifunctional electrocatalyst for overall water splitting, holding grand potential as a cost‐effective and efficient electrocatalyst in practical energy devices.

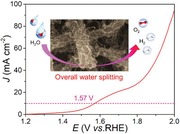

Due to the ever‐increasing global energy crisis and severe environmental pollution caused by the excessive consumption of traditional fossil fuel, it is indubitably paramount to explore eco‐friendly and sustainable energy sources. Molecular hydrogen (H_2_) has long been recognized as an ideal renewable energy carrier thanks to its high gravimetric energy density (≈142 MJ kg^−1^), superior utilization efficiency, and eco‐friendly nature.^1^, ^2^, ^3^ Electrochemical water splitting represents one of the most attractive and competitive technologies for the realization of sustainable hydrogen production because of its zero‐carbon emission advantage.^4^, ^5^, ^6^, ^7^ The water electrolysis involves two half reactions, hydrogen evolution reaction (HER) at cathode and oxygen evolution reaction (OER) at anode, both of which require high‐efficiency electrocatalysts to lower down the reaction overpotentials and thus expedite the reaction kinetics.^8^, ^9^ To date, Pt and Ir/Ru oxides are acknowledged as the state‐of‐the‐art electrocatalysts for the HER and the OER, respectively. However, the extreme scarcity, high cost, and inferior stability of these precious metals severely impeded the prevailing commercialization of water splitting devices.^10^, ^11^, ^12^ Therefore, the exploitation of nonprecious alternative electrocatalysts with cost effectiveness, high activity, and long‐term stability becomes greatly imperative. Particularly, the development of efficient bifunctional electrocatalysts that are capable of catalyzing both HER and OER simultaneously in the same electrolyte is extremely desired for the purpose of simplification of electrochemical system and reduction of production cost.

Accordingly, considerable research interests have been paid to searching efficient bifunctional electrocatalysts based on earth‐abundance 3d transition metal (TM = Fe, Co, Ni, and Mn, etc.) nanomaterials and their diverse derivatives, including multicomponent alloys,^13^, ^14^ chalcogenides,^15^, ^16^ carbides,^17^, ^18^ phosphides,^19^, ^20^ oxides/hydroxides,^21^, ^22^ nitrides,^23^, ^24^ and so on. Among these promising alternatives, Ni‐based alloys have garnered enormous attention due to the good corrosion resistance, high electrical conductivity, and suitable d‐electron configuration.^25^, ^26^, ^27^ Unfortunately, their electrocatalytic activity is still inferior to benchmark noble‐metal‐based catalysts and the stability needs to be further improved for the long‐term operation. One of the effective strategies to boost the electrocatalytic activity is to tune the catalyst's composition through the incorporation of second foreign atoms, modulating the electronic structure of the electrocatalytically active center and thus accelerating the activation of reactants.^28^, ^29^ On the other hand, immobilizing Ni‐based alloys onto conductive N‐doped nanocarbon can effectively increase the overall conductivity, protect the TMs from chemical corrosion, and suppress the aggregation/detachment of active species due to the confinement effect.^30^, ^31^, ^32^ Moreover, the coupling synergistic effect between the active sites and carbon support also contributes to enhancement of electrocatalytic activity. Considering the hierarchical branched architectures, especially the 1D/1D subunit coupled structures, can greatly enlarge the exposure of active sites and favor the mass diffusion/charge transport during the electrocatalytic reactions, and direct immobilization of Ni‐based alloys onto 1D/1D constructed hierarchical branched carbon substrate would be a reliable approach to overcome the shortcomings of insufficient activity and poor stability of Ni‐based catalysts for water electrolysis.^33^, ^34^, ^35^ For the construction of 1D/1D coupled hierarchical architecture, in situ growth of radial “branches” on axial “trunk” support could significantly strengthen the connectivity of the “branches” and the “trunk” support, thus greatly enhancing the mechanical robustness. Additionally, the seamless contact between the “branches” and the “trunk” substrate dramatically reduces the charge transfer resistance, forming good conductive networks. Therefore, in situ growth of “branches” on “trunk” support can remarkably enhance the reaction kinetics and catalyst's stability. However, it remains grand challenging so far to develop a facile and efficient synthetic approach to achieve the firm immobilization of Ni‐based alloy nanoparticles onto hierarchical branched carbon substrate as a bifunctional electrocatalyst for overall water splitting.

Motivated by the above considerations, we herein developed a simple and scalable electrospinning–pyrolysis strategy to directly immobilize uniform Ni_3_Co alloy nanoparticles onto in situ formed N‐doped carbon‐nanotube‐grafted carbon nanofibers (Ni_1.5_Co_0.5_ @ N‐C NT/NFs). The rational integration of Ni_3_Co nanoparticles, N‐doped CNTs, and N‐doped CNFs into hierarchical branched architectures renders the formed hybrid catalyst with abundant well‐dispersed accessible active sites, efficient electron transfer paths, facilitated mass diffusion, and reinforced mechanical strength. All these advantageous features synergistically contribute to the enhancement of both HER and OER activities in alkaline medium. The optimized Ni_1.5_Co_0.5_ @ N‐C NT/NFs catalyst delivers a current density of 10 mA cm^−2^ at overpotentials of 114 and 243 mV for HER and OER, respectively. What is more, when assembled into a two‐electrode alkaline electrolyzer for overall water splitting, it also shows a low potential requirement (1.57 V at 10 mA cm^−2^) and remarkable stability (at least 27 h), surpassing most of the previously reported nonprecious metal bifunctional catalysts and demonstrating potential applications in practical large‐scale H_2_ production.

As schematically illustrated in **Figure** **1**, the hierarchical structured Ni_1.5_Co_0.5_ @ N‐C NT/NFs were fabricated through a scalable electrospinning technique, followed by pyrolysis under a reducing atmosphere. In brief, a homogenous *N*,*N*‐dimethylformamide solution containing 1.5 mmol of Ni(NO_3_)_2_, 0.5 mmol of Co(NO_3_)_2_, and poly(vinylpyrrolidone) (PVP) was first electrospun into light‐pink fibrous membrane. During the electrospinning process, Ni^2+^ and Co^2+^ can be uniformly distributed within the PVP nanofibers to form Ni^2+^/Co^2+^/PVP composite nanofibers. Scanning electron microscopy (SEM) images (Figure S1, Supporting Information) indicate that the as‐spun Ni^2+^/Co^2+^/PVP fibrous membrane is composed of uniform and smooth nanofibers, which are randomly aligned and interconnected with each other to form continuous fiber networks. The average diameter of these nanofibers is identified to be ≈300 nm and the length is up to dozens of micrometers. After stabilization at 200 °C in air, the resultant nanofibers were further pyrolyzed at 700 °C in an Ar/H_2_ atmosphere. When annealed in such strongly reducing atmosphere, the PVP nanofibers were transformed into N‐doped carbon nanofibers, accompanied by the reduction of metal ions into nanosized metallic Ni_3_Co nanoparticles uniformly dispersed throughout the nanofibers. Simultaneously, the surface‐residing Ni_3_Co nanoparticles also served as nanocatalysts for the in situ growth of carbon nanotubes from the nanofiber surface, resulting in the formation of hierarchical branch‐like architectures constructed by Ni_3_Co nanoparticle encapsulated carbon‐nanotube‐grafted nanofibers. It is worth mentioning that the in situ growth manner of Ni_3_Co nanoparticles and carbon nanotubes on the nanofibers can greatly strengthen the connection between active sites and substrate, which is beneficial for charge transport and also prevents the detachment of active sites.

**Figure 1:**
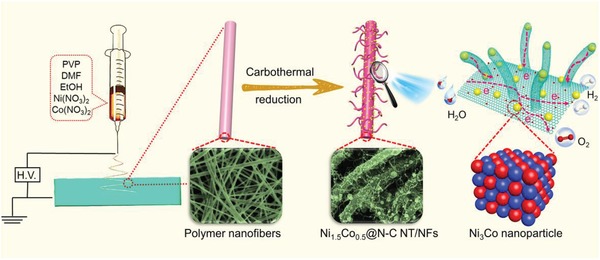
Schematic illustration of the fabrication procedure of the Ni_1.5_Co_0.5_ @ N‐C NT/NFs.

As disclosed by the SEM images shown **Figure** **2**a–c, the as‐fabricated Ni_1.5_Co_0.5_ @ N‐C NT/NFs perfectly inherit the fibrous morphology yet with a relatively rough surface and shrunk diameter of ≈150 nm. A closer observation demonstrates that the hierarchical Ni_1.5_Co_0.5_ @ N‐C NT/NFs are decorated by numerous uniform Ni_3_Co nanoparticles and wrapped with plenty of flexible carbon nanotubes, featuring as hierarchically branched architectures. It is noteworthy that the carbon nanotubes seamlessly rooted in the nanofiber backbones serve not only as spacers to effectively prevent the agglomeration of carbon nanofibers, but also as current collectors to improve charge transfer. Therefore, such nanotube/nanofiber integrated structure is favorable for mass diffusion/electron transfer and permeation of electrolyte during the electrocatalysis process. Transmission electron microscopy (TEM) images (Figure 2d,e) further verify that a mass of Ni_3_Co nanoparticles with an average grain size of 15.2 nm (Figure S2, Supporting Information) are evenly dispersed in the hierarchically branched Ni_1.5_Co_0.5_ @ N‐C NT/NFs. The immobilization of Ni_3_Co nanoparticles in nanofiber matrix not only prevents the metal nanoparticles from detachment, migration, and self‐aggregation, but also enhances the conductivity of the obtained nanohybrids. Additionally, the embedded Ni_3_Co nanoparticles may exhibit high corrosion resistance in a harsh electrolyte due to the carbon protection. The TEM images also display a great number of nanotubes germinating from the nanofiber support, forming accessible gaps between the nanotube tentacles, and thus significantly increasing the surface area. A magnified TEM image (Figure 2f) shows that the Ni_3_Co nanoparticles are encased into the top of carbon nanotubes, suggesting a tip‐growth mechanism.^36^, ^37^ The carbon nanotubes have outer diameters in the range of 10–20 nm and inner diameters around 5 nm, demonstrating a multi‐walled feature. The high‐resolution TEM (HRTEM) image (Figure 2g) further verifies the highly graphitized multi‐walled carbon nanotubes with an interlayer spacing of 0.36 nm, corresponding to the (002) reflection of graphitic carbon. Notably, the orientation of graphitic carbon layers is not parallel to the axial direction of nanotube, providing more defects and edges in the carbon nanotubes.^38^ The HRTEM image of the Ni_3_Co nanoparticle (Figure 2h) exhibits the well‐resolved fringe spacing of 0.206 nm, ascribing to (111) plane of Ni_3_Co alloy. The elemental mapping analysis (Figure 2i) suggests the coexistence and homogenous distribution of Ni, Co, N, and C within the entire hierarchically branched structure.

**Figure 2:**
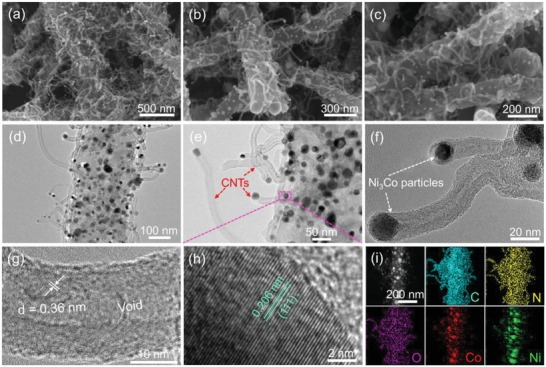
Morphological examination of the as‐obtained Ni_1.5_Co_0.5_ @ N‐C NT/NFs. a–c) SEM images, d–f) TEM images, g–h) HRTEM images, and i) HADDF‐STEM image and elemental mapping images.

As exhibited in the X‐ray diffraction (XRD) pattern in **Figure** **3**a, three prominent diffraction peaks positioned at 44.3^o^, 51.6^o^, and 76.1^o^ can be well indexed to the (111), (200), and (220) facets of face‐centered cubic Ni_3_Co (JCPDS No. 01‐074‐5694), respectively. The broad and inconspicuous peak located at ≈26° can be ascribed to the (002) plane of graphitic carbon. Energy‐dispersive X‐ray (EDX) spectrum shown in Figure 3b suggests that the atomic ratio of Ni/Co in the obtained Ni_1.5_Co_0.5_ @ N‐C NT/NFs is determined as 74.07:25.93, being in consistence with the feeding molar ratio (Ni/Co = 3/1). The graphitization feature of carbon in the Ni_1.5_Co_0.5_ @ N‐C NT/NF sample is investigated by Raman spectrum (Figure 3c). Two distinctive peaks centered at 1347 and 1594 cm^−1^ correspond to D band (disordered or defect carbon) and G band (graphitized sp^2^ carbon), respectively. The relative peak intensity ratio (*I*_D_/*I*_G_) is calculated to be 0.96, signifying a high graphitization degree of the carbon support and thus an excellent electronic conductivity of the nanohybrid. According to the thermogravimetric analysis (TGA) curve in Figure 3d, the weight percentage of Ni_3_Co nanoparticles in the resultant Ni_1.5_Co_0.5_ @ N‐C NT/NFs is determined to be 40%. The textural property and pore feature of the Ni_1.5_Co_0.5_ @ N‐C NT/NFs were characterized by N_2_ sorption measurement. As displayed in Figure 3e, the isotherms for Ni_1.5_Co_0.5_ @ N‐C NT/NFs can be categorized as type‐IV with a distinct hysteresis loop located in the relative pressure (*p*/*p*_0_) range of 0.4–0.9, implying the *meso*‐porous feature of the formed Ni_1.5_Co_0.5_ @ N‐C NT/NFs. Furthermore, the steep uptake of N_2_ at low relative pressure (0–0.015) suggests the existence of micropores, which are caused by the decomposition of the polymer nanofibers during the pyrolysis process. Therefore, the coexistence of micropores and *meso*‐pores endows the Ni_1.5_Co_0.5_  @  N‐C NT/NF sample with a high Brunauer–Emmett–Teller surface area of 425.8 m^2^ g^−1^, which is significantly higher than those of previously reported electrospinning‐derived 1D nanomaterials.^39^, ^40^, ^41^, ^42^ The pore‐size distribution curve (Figure 3f) suggests the average pore size in Ni_1.5_Co_0.5_  @  N‐C NT/NFs is ≈3.9 nm, which agrees well with the aforementioned N_2_ isotherms.

**Figure 3:**
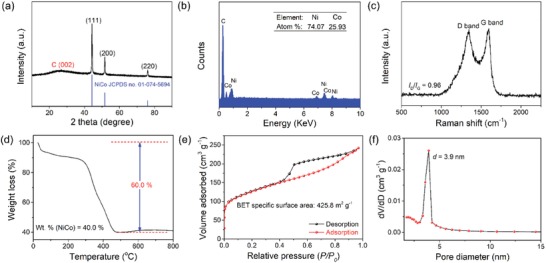
Compositional characterization of the fabricated Ni_1.5_Co_0.5_ @ N‐C NT/NFs. a) XRD pattern, b) EDX spectrum, c) Raman spectrum, d) TGA curve, e) N_2_ adsorption–desorption isotherms, and f) the pore‐size distribution curve.

The surface chemistry and valence state of each constituent element in Ni_1.5_Co_0.5_  @  N‐C NT/NFs are examined by X‐ray photoelectron spectroscopy (XPS) technique. As disclosed by the XPS survey spectrum (**Figure** **4**a), Ni, Co, C, N, and O can be detected on the surface of Ni_1.5_Co_0.5_  @  N‐C NT/NFs. The N element originates from the pyrolysis of PVP, and O element stems from the surface oxygen‐containing functional groups. The high‐resolution Ni 2p spectrum (Figure 4b) exhibits two predominant peaks located at ≈852.7 and 870.0 eV, which correspond to zero‐valence state metallic Ni. The relatively weak peaks positioned at 855.8 and 873.6 eV can be ascribed to Ni^2+^ 2p_3/2_ and Ni^2+^ 2p_1/2_, respectively, due to the partial oxidation of the sample exposed to air. It is noteworthy that the Ni^2+^ species can serve as active sites for water dissociation involved in the HER process in an alkaline medium.^43^ Likewise, the high‐resolution Co 2p spectrum (Figure 4c) can be well deconvoluted into metallic Co (778.4 and 794.5 eV) and Co^2+^ (783.0 and 802.2 eV). The high‐resolution C 1s spectrum (Figure 4d) can be well fitted into three peaks located at 284.4, 285.3, and 289.1 eV, which are assigned to C—C, C—N, and C=O, respectively. The high‐resolution spectrum of O 1s (Figure 4e) reveals the presence of metal—O bond (531.3 eV), O—C—O (532.4 eV), and C—OH (533.8 eV). For high‐resolution N 1s spectrum (Figure 4f), the four well‐fitted peaks correspond to pyridinic‐N (398.4 eV), pyrrolic‐N (400.3 eV), graphitic‐N (401.0 eV), and oxidized‐N (403.9 eV), respectively. The successful incorporation of N atoms into the formed Ni_1.5_Co_0.5_  @  N‐C NT/NFs could modulate the local electronic structure, enhance the electrical conductivity, and create abundant defects/vacancies, which is favorable to improve the electrocatalytic performance.^44^

**Figure 4:**
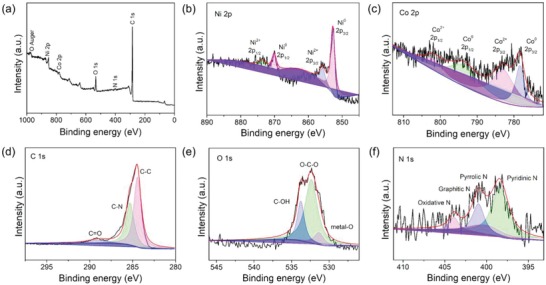
a) Typical XPS survey spectrum and b–f) high‐resolution spectra for the Ni 2p (b), Co 2p (c), C 1s (d), O 1s (e), and N 1s (f) regions of the resultant Ni_1.5_Co_0.5_ @ N‐C NT/NFs sample.

It is noticeable that the synthetic parameters, including pyrolysis temperature, feeding ratio, and amount of metal precursors, exert considerable influence on the structural feature and composition of the resultant products. For the standard synthesis of Ni_1.5_Co_0.5_  @  N‐C NT/NF sample, the pyrolysis temperature is deliberately set at 700 °C, and the Ni^2+^ and Co^2+^ sources are fixed as 1.5 and 0.5 mmol, respectively. When the pyrolysis temperature is lowered to 600 °C while the other synthetic parameters keep unchanged, only very sparse carbon nanotubes can be occasionally observed from the surface of the nanoparticle‐embedded carbon nanofibers (Figure S3a,b, Supporting Information). The increment of pyrolysis temperature to 650 °C leads to the lush in situ growth of carbon nanotubes on the surface of nanoparticle‐embedded carbon nanofibers (Figure S3c,d, Supporting Information). However, the distribution density and aspect ratio of the generated carbon nanotubes are remarkably less than those observed on the counterpart obtained in 700 °C (Figure 2). As the pyrolysis temperature is further raised to 750 °C, the Ni_3_Co nanoparticles suffer from a serious agglomeration and thus loss the catalytic capability toward the growth of carbon nanotubes, leading to the formation of fragmented nanofibers embedded with coarsened Ni_3_Co nanoparticles (Figure S3e,f, Supporting Information). Therefore, the calcination temperature of 700 °C represents the optimal temperature to achieve the nanotube/nanofiber integrated hierarchically branched architectures. Keeping the total amount of metal precursors at 2.0 mmol yet varying their feeding ratios also dramatically changes the morphology of the fabricated products. When only 2.0 mmol of Ni(NO_3_)_2_ is involved as the sole metal precursor, some slim carbon nanotubes sprout from the Ni nanoparticle‐embedded carbon nanofibers (denoted as Ni @ N‐C NT/NFs; Figure S4a–c, Supporting Information). While the only introduction of 2.0 mmol of Co(NO_3_)_2_ results in the generation of Co nanoparticle‐embedded nanofibers (abbreviated as Co @ N‐CNFs; Figure S4d–f, Supporting Information). Additionally, metal precursors containing either 0.5 mmol of Ni^2+^/1.5 mmol of Co^2+^ or 1.0 mmol of Ni^2+^/1.0 mmol of Co^2+^ give rise to the formation of nanoparticle‐embedded carbon nanofibers without the decoration of carbon nanotubes (denoted as Ni_0.5_Co_1.5_  @  N‐CNFs and Ni_1.0_Co_1.0_  @  N‐CNFs, respectively; Figure S5, Supporting Information). As the total amount of metal precursors is decreased to 1.0 mmol of Ni^2+^/0.33 mmol of Co^2+^, the obtained product features as a the nanotube/nanofiber coupled hierarchically branched structure (denoted as Ni_1.0_Co_0.33_  @  N‐C NT/NFs; Figure S6a,b, Supporting Information). However, the density of the surface carbon nanotubes is significantly less than that observed from the standard sample, due to the fewer growth sites for carbon nanotubes caused by the insufficient metal sources. On the contrary, the excessive feeding of metal sources of 2.0 mmol of Ni^2+^/0.67 mmol of Co^2+^ brings about the severely aggregated Ni_3_Co nanoparticle‐embedded nanofibers (denoted as Ni_2.0_Co_0.67_  @  N‐CNFs; Figure S6c,d, Supporting Information). Therefore, the optimal pyrolysis temperature, appropriate feeding ratio, and total amount of metal precursors are a prerequisite for the generation of Ni_3_Co nanoparticle‐encased nanotube/nanofiber integrated hierarchical architecture.

The encapsulation of uniform Ni_3_Co nanoparticles into N‐doped carbon nanotube/nanofiber integrated hierarchical architecture makes the as‐prepared Ni_1.5_Co_0.5_  @  N‐C NT/NFs promising as an earth‐abundant electrocatalyst. The electrocatalytic performance of the fabricated Ni_1.5_Co_0.5_  @  N‐C NT/NF catalyst for HER and OER was evaluated by recording linear sweep voltammetry (LSV) curves in 0.1 m KOH solution using a standard three‐electrode configuration. In order to highlight the superiority of the prepared Ni_1.5_Co_0.5_  @  N‐C NT/NFs, we first studied the HER and OER activities of a set of control samples, including various samples achieved at different pyrolysis temperature, feeding ratio, and total amount of metal precursors (Figures S3–S6, Supporting Information). Obviously, the pyrolysis temperature, feeding ratio, and total amount of metal precursors had a remarkable impact on the electrocatalytic performance. As displayed in Figure S7 (Supporting Information), the standard Ni_1.5_Co_0.5_  @  N‐C NT/NF sample exhibits the highest HER and OER activities among all control samples investigated herein, representing the most optimized catalyst due to the hierarchical branched structure and appropriate metal loading amount. Additionally, Ni @ N‐C NT/NFs, Co @ N‐CNFs, bare N‐doped carbon nanofibers (denoted as N‐CNFs; Figure S8, Supporting Information), and commercial noble metal‐based catalysts (20% Pt/C and RuO_2_) were also evaluated as references under the identical test condition. The ohmic potential drop (*iR*) losses were corrected, as shown in Figure S9 (Supporting Information). **Figure** **5**a displays the typical *iR*‐corrected LSV curves of HER for different samples with a scan rate of 5 mV s^−1^. As expected, the commercial Pt/C catalyst exhibits the highest HER activity with a near‐zero onset potential versus reversible hydrogen electrode (RHE) and large current density, while the bare N‐CNFs show almost negligible HER activity. As compared with Ni @ N‐C NT/NFs and Co @ N‐CNFs, the Ni_1.5_Co_0.5_  @  N‐C NT/NF sample exhibits a fast‐rising cathodic current response with the increasing of applied negative potentials and thus delivers a remarkable HER activity, further emphasizing the superiority of hierarchically branched architecture and synergy between Ni and Co. As displayed in Figure 5b, the Ni_1.5_Co_0.5_  @  N‐C NT/NF catalyst requires an overpotential of 114 mV to afford a current density of 10 mA cm^−2^, which is higher than that of commercial Pt/C (46 mV) but much smaller than that of Ni @ N‐C NT/NFs (127 mV) or Co @ N‐CNFs (395 mV). Additional, Tafel plots (Figure 5c) were further adopted to investigate the HER kinetics of different catalysts and a small value of Tafel slope generally suggests favorable electrocatalytic kinetics. Unquestionably, the commercial Pt/C catalyst exhibits the lowest Tafel slope of 54 mV dec^−1^. The Tafel slope of standard Ni_1.5_Co_0.5_  @  N‐C NT/NFs is measured to be 117 mV dec^−1^, significantly lower than that of Ni @ N‐C NT/NFs (232 mV dec^−1^) or Co @ N‐CNFs (301 mV dec^−1^). The Tafel slope of Ni_1.5_Co_0.5_  @  N‐C NT/NFs also manifests that the HER process proceeds a Volmer–Heyrovsky pathway with the Volmer reaction as the rate‐determining step. Notably, the HER activity of current Ni_1.5_Co_0.5_  @  N‐C NT/NF catalyst is comparable or even superior to many other reported nonprecious catalysts in terms of small overpotential and low Tafel slope (Table S1, Supporting Information).

**Figure 5:**
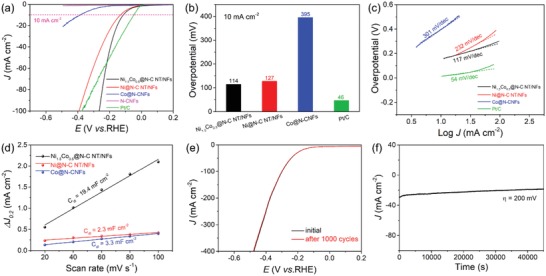
Evaluation of HER performance of different samples in 0.1 m KOH solution. a) LSV polarization curves. b) Required overpotentials at a current density of 10 mA cm^−2^. c) Tafel plots. d) Capacitive current at 0.20 V as a function of scan rate. e) LSV curves of Ni_1.5_Co_0.5_ @ N‐C NT/NF sample before and after 1000 cycles. f) The chronoamperometric response of Ni_1.5_Co_0.5_ @ N‐C NT/NFs at an overpotential of 200 mV.

As well documented, the electrochemically active surface area of nanocatalysts with similar compositions is linearly proportional to their electrochemical double‐layer capacitance (*C*_dl_) values. Derived from cyclic voltammograms (CVs) with different scan rates from 20 to 100 mV s^−1^ within a nonfaradic potential region of 0.15–0.25 V in 0.1 m KOH solution (Figure S10, Supporting Information), the *C*_dl_ value of 19.4 mF cm^−2^ on the standard Ni_1.5_Co_0.5_  @  N‐C NT/NFs is considerably higher than those on Ni @ N‐C NT/NFs (2.3 mF cm^−2^) or Co @ N‐CNFs (3.3 mF cm^−2^), as depicted in Figure 5d. The high *C*_dl_ value of Ni_1.5_Co_0.5_  @  N‐C NT/NFs signifies the enriched exposure of active sites for HER. The stability of Ni_1.5_Co_0.5_  @  N‐C NT/NF sample was examined by continuous CV scanning in the potential range of −0.2–0.2 V for 1000 cycles and chronoamperometry test. After the cycling test, the catalyst exhibits an LSV curve almost overlapped with the initial one with negligible negative shift of overpotential (Figure 5e). Additionally, the *i*–*t* curve (Figure 5f) again verifies that the current density maintains quite stable even after a period of 45 000 s at a constant overpotential of 200 mV. Moreover, TEM image (Figure S11, Supporting Information) confirms that the characteristic nanotube/nanofiber integrated hierarchical architecture of Ni_1.5_Co_0.5_  @  N‐C NT/NFs is well preserved and Ni_3_Co nanoparticles are still firmly embedded within the carbon support after long‐term electrolysis, indicating their outstanding mechanical robustness.

The OER catalytic performance of the Ni_1.5_Co_0.5_  @  N‐C NT/NF sample was also appraised in 0.1 m O_2_‐saturated KOH solution. **Figure** **6**a shows the LSV curves after *iR* compensation of different samples. The standard Ni_1.5_Co_0.5_  @  N‐C NT/NF sample affords the highest OER activity with the lowest overpotential of 243 mV to deliver a current density of 10 mA cm^−2^, while Ni @ N‐C NT/NFs, Co @ N‐CNFs, and commercial RuO_2_ catalyst require much larger overpotentials of 630, 436, and 351 mV, respectively (Figure 6b). Figure 6c illustrates that the Tafel slope of Ni_1.5_Co_0.5_  @  N‐C NT/NFs is determined to be 103 mV dec^−1^, which is smaller than those of Ni @ N‐C NT/NFs (156 mV dec^−1^) and Co @ N‐CNFs (105 mV dec^−1^), implying faster OER reaction kinetics. Although the commercial RuO_2_ catalyst exhibits a smaller Tafel slope of 67 mV dec^−1^, the overpotential on RuO_2_ catalyst is remarkably larger than that on Ni_1.5_Co_0.5_  @  N‐C NT/NFs (351 mV vs 243 mV) at a current density of 10 mA cm^−2^. The OER performance of Ni_1.5_Co_0.5_  @  N‐C NT/NFs also outperforms or can compete with previously reported noble metal‐free catalysts (Table S2, Supporting Information). The durability of the Ni_1.5_Co_0.5_  @  N‐C NT/NF catalyst toward the OER was further investigated by CV scanning in the potential range of 1.2–1.4 V for 1000 cycles and long‐term chronoamperometry test. As proved by the almost identical LSV plots before and after continuous 1000 cycles (Figure 6d), the Ni_1.5_Co_0.5_  @  N‐C NT/NFs demonstrate a superior electrochemical OER stability, which is again certified by the stable *i*–*t* curve (inset of Figure 6d) performed at 1.60 V versus RHE for 45 000 s. Moreover, TEM images (Figure S12, Supporting Information) indicate that the structural feature of Ni_1.5_Co_0.5_  @  N‐C NT/NFs can also be well retained after the stability test. Encouraged by the outstanding HER and OER activities of the standard Ni_1.5_Co_0.5_  @  N‐C NT/NF catalyst, we then equipped a two‐electrode electrolyzer using Ni_1.5_Co_0.5_  @  N‐C NT/NF as a bifunctional catalyst to examine its overall water splitting performance in 1.0 m KOH electrolyte (inset of Figure 6e). The Ni_1.5_Co_0.5_  @  N‐C NT/NF catalyst exhibits an impressive water splitting performance with a cell voltage of only 1.57 V to deliver a current density of 10 mA cm^−2^, exceeding most of the reported nonprecious metal bifunctional catalysts (Table S3, Supporting Information). A great number of gas bubbles of H_2_ and O_2_ can be appreciably produced from the cathode and anode, respectively, as the applied potential increases (inset of Figure 6e). Moreover, the Ni_1.5_Co_0.5_  @  N‐C NT/NF‐assembled electrolyzer maintains a pretty stable current density for 27 h at a constant applied potential of 1.57 V (Figure 6f), revealing the extraordinary stability of the Ni_1.5_Co_0.5_  @  N‐C NT/NF catalyst toward overall water splitting. As evidenced by the TEM images (Figure S13a,b, Supporting Information), the 1D/1D integrated hierarchical structure of the catalyst after the long‐term stability test could be well retained and Ni_3_Co nanoparticles are well dispersed thanks to the immobilization effect of carbon scaffold. The HRTEM image (Figure S13c, Supporting Information) of an individual Ni_3_Co nanoparticle further confirms that nanoparticles are firmly dispersed within the carbon matrix. The HRTEM images (Figure S13d–f, Supporting Information) of a single carbon nanotube display that the highly graphitized multi‐walled carbon nanotube still roots in the carbon nanofiber substrate. All these results unambiguously affirm the mechanical stability of the obtained Ni_1.5_Co_0.5_  @  N‐C NT/NFs after long‐term stability test. Furthermore, XPS was utilized to characterize the surface chemistry of the catalyst after stability test. The high‐resolution Ni 2p spectrum (Figure S14a, Supporting Information) suggests the generation of Ni^3+^ species, and the Co 2p spectrum (Figure S14b, Supporting Information) demonstrates the presence of Co^2+^ species after the stability test. These results suggest that the surface of the catalyst is partially oxidized during the long‐term electrolysis process. The outstanding activity and durability make the Ni_1.5_Co_0.5_  @  N‐C NT/NF catalyst a potential alternative to precious electrocatalysts for cost‐effective and energy‐efficient water splitting.

**Figure 6:**
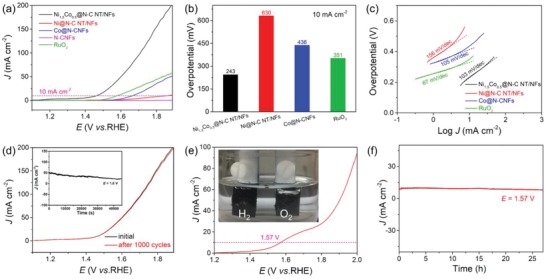
Comparison of electrocatalytic OER performance of different samples. a) LSV polarization curves. b) Required overpotentials at a current density of 10 mA cm^−2^. c) Tafel plots. d) LSV curves of Ni_1.5_Co_0.5_ @ N‐C NT/NFs before and after 1000 cycles and the inset shows the *i*–*t* curve at a potential of 1.6 V. e) Polarization curve of Ni_1.5_Co_0.5_ @ N‐C NT/NFs for overall water splitting in 1.0 m KOH and the inset shows the H_2_ and O_2_ evolution during the electrolysis process. f) Chronoamperometry measurement of Ni_1.5_Co_0.5_ @ N‐C NT/NFs for overall water splitting at a voltage of 1.57 V.

The extraordinary electrocatalytic activity and stability of the fabricated Ni_1.5_Co_0.5_  @  N‐C NT/NF catalyst can be mainly ascribed to the elaborate architectural superiority and compositional synergy. (1) The firm immobilization of Ni_3_Co nanoparticles into graphitic carbon matrix can effectively prevent the nanoparticles from undesirable aggregation, chemical leaching, and detachment during the electrocatalytic process, ensuring the excellent mechanical stability.^45^ (2) As a conductive current collector, the integrated N‐C NT/NF hierarchical branched architecture provides a facilitated electron highway, accelerating the electron transfer during the catalytic process. Additionally, such branched structure with more exposure of active sites is beneficial for the penetration of electrolyte and release of generated H_2_ and O_2_ bubbles during the electrolysis. (3) The incorporation of N dopant into carbon matrix may modulate the electronic structure of neighboring carbon, which can effectively tune the adsorption free energy of the H atom and also facilitate the water oxidation process.^46^ Moreover, the N doping into carbon matrix can create additional active sites and improve chemical and thermal stability. (4) The synergistic effect between Ni and Co in the formed Ni_3_Co alloy might modulate the electronic structure of the active sites and thus alter the chemisorption energies of reaction intermediates, affording an optimal catalytic activity.^47^, ^48^ All in all, thanks to the aforementioned architectural and compositional advantageous features, the Ni_1.5_Co_0.5_ @ N‐C NT/NF catalyst is demonstrated as an exceptional bifunctional electrocatalyst toward the overall water splitting.

In conclusion, we have elaborately designed a hierarchical branched architecture composed of uniform Ni_3_Co alloyed nanoparticles immobilized in in situ formed N‐doped carbon‐nanotube‐grafted nanofibers through a feasible and scalable electrospinning–pyrolysis strategy. The intimate integration of Ni_3_Co alloyed nanoparticles, N‐doped CNTs, and N‐doped CNFs into hierarchical branched structure endows the optimized Ni_1.5_Co_0.5_ @ N‐C NT/NF catalyst with enriched active sites, favorable electron configuration, and facilitated diffusion kinetics. As such, the formed Ni_1.5_Co_0.5_ @ N‐C NT/NF catalyst exhibits extraordinary performance for both HER and OER, which is corroborated by the low overpotentials of 114 and 243 mV at a current density of 10 mA cm^−2^, respectively, under alkaline condition. When used as a bifunctional electrocatalyst for overall water splitting, the constructed two‐electrode electrolyzer only requires a cell voltage of 1.57 V to afford a current density of 10 mA cm^−2^, outperforming most of the previously reported nonprecious metal bifunctional catalysts. It also displays remarkable stability after a continuous operation for 27 h without noticeable attenuation. This work may shed new insight into the rational design and facile fabrication of high‐efficiency transition‐metal‐based bifunctional electrocatalysts as promising alternatives for low‐cost and large‐scale water electrolysis.

## Conflict of Interest

The authors declare no conflict of interest.

## Supporting information

Supporting Information

## References

[1] G. Zhao , K. Rui , S. X. Dou , W. Sun , Adv. Funct. Mater. 2018, 28, 1803291.

[2] M.‐Q. Wang , C. Tang , C. Ye , J. Duan , C. Li , Y. Chen , S.‐J. Bao , M. Xu , J. Mater. Chem. A 2018, 6, 14734.

[3] W. Cui , Q. Liu , Z. Xing , A. M. Asiri , K. A. Alamry , X. Sun , Appl. Catal., B 2015, 164, 144.

[4] T. Ouyang , Y. Q. Ye , C. Y. Wu , K. Xiao , Z. Q. Liu , Angew. Chem., Int. Ed. 2019, 58, 4923. 10.1002/anie.201814262 30635963

[5] T. Tang , W. J. Jiang , S. Niu , N. Liu , H. Luo , Y. Y. Chen , S. F. Jin , F. Gao , L. J. Wan , J. S. Hu , J. Am. Chem. Soc. 2017, 139, 8320. 28535047 10.1021/jacs.7b03507

[6] J. Lai , B. Huang , Y. Chao , X. Chen , S. Guo , Adv. Mater. 2019, 31, 1805541. 10.1002/adma.201805541 30417441

[7] X. Yu , Z. Y. Yu , X. L. Zhang , Y. R. Zheng , Y. Duan , Q. Gao , R. Wu , B. Sun , M. R. Gao , G. Wang , S. H. Yu , J. Am. Chem. Soc. 2019, 141, 7537. 31017425 10.1021/jacs.9b02527

[8] Y. Li , J. Yin , L. An , M. Lu , K. Sun , Y. Q. Zhao , D. Gao , F. Cheng , P. Xi , Small 2018, 14, 1801070. 10.1002/smll.201801070 29808557

[9] L. Huang , D. Chen , G. Luo , Y. R. Lu , C. Chen , Y. Zou , C. L. Dong , Y. Li , S. Wang , Adv. Mater. 2019, 31, 1901439. 10.1002/adma.201901439 31148279

[10] C. Huang , T. Ouyang , Y. Zou , N. Li , Z.‐Q. Liu , J. Mater. Chem. A 2018, 6, 7420.

[11] G. Huang , Z. Xiao , R. Chen , S. Wang , ACS Sustainable Chem. Eng. 2018, 6, 15954.

[12] M. Li , Y. Zhu , H. Wang , C. Wang , N. Pinna , X. Lu , Adv. Energy Mater. 2019, 9, 1803185.

[13] F. Qin , Z. Zhao , M. K. Alam , Y. Ni , F. Robles‐Hernandez , L. Yu , S. Chen , Z. Ren , Z. Wang , J. Bao , ACS Energy Lett. 2018, 3, 546.

[14] X. Zhang , H. Xu , X. Li , Y. Li , T. Yang , Y. Liang , ACS Catal. 2015, 6, 580.

[15] Y. Yang , K. Zhang , H. Lin , X. Li , H. C. Chan , L. Yang , Q. Gao , ACS Catal. 2017, 7, 2357.

[16] Q. Li , D. Wang , C. Han , X. Ma , Q. Lu , Z. Xing , X. Yang , J. Mater. Chem. A 2018, 6, 8233.

[17] H. Lin , W. Zhang , Z. Shi , M. Che , X. Yu , Y. Tang , Q. Gao , ChemSusChem 2017, 10, 2597. 28371425 10.1002/cssc.201700207

[18] M. A. R. Anjum , M. H. Lee , J. S. Lee , ACS Catal. 2018, 8, 8296.

[19] C. Tang , R. Zhang , W. Lu , L. He , X. Jiang , A. M. Asiri , X. Sun , Adv. Mater. 2017, 29, 1602441. 10.1002/adma.201602441 27797162

[20] T. Liu , L. Xie , J. Yang , R. Kong , G. Du , A. M. Asiri , X. Sun , L. Chen , ChemElectroChem 2017, 4, 1840.

[21] G. Chen , T. Wang , J. Zhang , P. Liu , H. Sun , X. Zhuang , M. Chen , X. Feng , Adv. Mater. 2018, 30, 1706279. 10.1002/adma.201706279 29349907

[22] Y. P. Zhu , T. Y. Ma , M. Jaroniec , S. Z. Qiao , Angew. Chem., Int. Ed. 2017, 56, 1324. 10.1002/anie.201610413 27900829

[23] Y. Gu , S. Chen , J. Ren , Y. A. Jia , C. Chen , S. Komarneni , D. Yang , X. Yao , ACS Nano 2018, 12, 245. 29257880 10.1021/acsnano.7b05971

[24] X. Jia , Y. Zhao , G. Chen , L. Shang , R. Shi , X. Kang , G. I. N. Waterhouse , L.‐Z. Wu , C.‐H. Tung , T. Zhang , Adv. Energy Mater. 2016, 6, 1502585.

[25] V. Vij , S. Sultan , A. M. Harzandi , A. Meena , J. N. Tiwari , W.‐G. Lee , T. Yoon , K. S. Kim , ACS Catal. 2017, 7, 7196.

[26] M. Y. Gao , C. Yang , Q. B. Zhang , J. R. Zeng , X. T. Li , Y. X. Hua , C. Y. Xu , P. Dong , J. Mater. Chem. A 2017, 5, 5797.

[27] S.‐W. Park , I. Kim , S.‐I. Oh , J.‐C. Kim , D.‐W. Kim , J. Catal. 2018, 366, 266.

[28] C. L. Huang , X. F. Chuah , C. T. Hsieh , S. Y. Lu , ACS Appl. Mater. Interfaces 2019, 11, 24096. 31185711 10.1021/acsami.9b05919

[29] H. Xu , J. Wei , M. Zhang , J. Wang , Y. Shiraishi , L. Tian , Y. Du , Nanoscale 2018, 10, 18767. 30276398 10.1039/c8nr05279d

[30] H. Sun , Y. Lian , C. Yang , L. Xiong , P. Qi , Q. Mu , X. Zhao , J. Guo , Z. Deng , Y. Peng , Energy Environ. Sci. 2018, 11, 2363.

[31] Z.‐Y. Wu , W.‐B. Ji , B.‐C. Hu , H.‐W. Liang , X.‐X. Xu , Z.‐L. Yu , B.‐Y. Li , S.‐H. Yu , Nano Energy 2018, 51, 286.

[32] Y. Cao , Y. Lu , E. H. Ang , H. Geng , X. Cao , J. Zheng , H. Gu , Nanoscale 2019, 11, 15112. 31368469 10.1039/c9nr05504e

[33] T. Li , G. Luo , K. Liu , X. Li , D. Sun , L. Xu , Y. Li , Y. Tang , Adv. Funct. Mater. 2018, 28, 1805828.

[34] H. Q. Fu , L. Zhang , C. W. Wang , L. R. Zheng , P. F. Liu , H. G. Yang , ACS Energy Lett. 2018, 3, 2021.

[35] Y. Zhao , J. Zhang , X. Guo , H. Fan , W. Wu , H. Liu , G. Wang , J. Mater. Chem. A 2017, 5, 19672.

[36] Z. Fan , J. Yan , L. Zhi , Q. Zhang , T. Wei , J. Feng , M. Zhang , W. Qian , F. Wei , Adv. Mater. 2010, 22, 3723. 20652901 10.1002/adma.201001029

[37] Y. Hou , S. Cui , Z. Wen , X. Guo , X. Feng , J. Chen , Small 2015, 11, 5940. 26449376 10.1002/smll.201502297

[38] J. Meng , C. Niu , L. Xu , J. Li , X. Liu , X. Wang , Y. Wu , X. Xu , W. Chen , Q. Li , Z. Zhu , D. Zhao , L. Mai , J. Am. Chem. Soc. 2017, 139, 8212. 28541686 10.1021/jacs.7b01942

[39] T. Li , Y. Lv , J. Su , Y. Wang , Q. Yang , Y. Zhang , J. Zhou , L. Xu , D. Sun , Y. Tang , Adv. Sci. 2017, 4, 1700226. 10.1002/advs.201700226 PMC5700636 29201620

[40] Z. Wang , J. Ang , B. Zhang , Y. Zhang , X. Y. D. Ma , T. Yan , J. Liu , B. Che , Y. Huang , X. Lu , Appl. Catal., B 2019, 254, 26.

[41] M. Wang , C. Zhang , T. Meng , Z. Pu , H. Jin , D. He , J. Zhang , S. Mu , J. Power Sources 2019, 413, 367.

[42] Q. Mo , W. Zhang , L. He , X. Yu , Q. Gao , Appl. Catal., B 2019, 244, 620.

[43] D. Das , S. Santra , K. K. Nanda , ACS Appl. Mater. Interfaces 2018, 10, 35025. 30244572 10.1021/acsami.8b09941

[44] W. Fu , E. Zhao , Z. Sun , X. Ren , A. Magasinski , G. Yushin , Adv. Funct. Mater. 2018, 28, 1801711.

[45] Z. Wang , W. Xu , X. Chen , Y. Peng , Y. Song , C. Lv , H. Liu , J. Sun , D. Yuan , X. Li , X. Guo , D. Yang , L. Zhang , Adv. Funct. Mater. 2019, 29, 1902875.

[46] T. Li , M. Li , M. Zhang , X. Li , K. Liu , M. Zhang , X. Liu , D. Sun , L. Xu , Y. Zhang , Y. Tang , Carbon 2019, 153, 364.

[47] Y. Yang , Z. Lin , S. Gao , J. Su , Z. Lun , G. Xia , J. Chen , R. Zhang , Q. Chen , ACS Catal. 2016, 7, 469.

[48] Q. Zhao , J. Yang , M. Liu , R. Wang , G. Zhang , H. Wang , H. Tang , C. Liu , Z. Mei , H. Chen , F. Pan , ACS Catal. 2018, 8, 5621.

