# Supplementary material for: Immobilization of Ni3Co Nanoparticles into N‐Doped Carbon Nanotube/Nanofiber Integrated Hierarchically Branched Architectures toward Efficient Overall Water Splitting
Source: Adv Sci (Weinh). 2019 Dec 1;7(1):1902371. doi: 10.1002/advs.201902371 (PMC6947496; doi:10.1002/advs.201902371)
Supplement: Supplementary file 1 — Supporting Information [file ADVS-7-1902371-s001.pdf]

Copyright WILEY-VCH Verlag GmbH & Co. KGaA, 69469 Weinheim, Germany, 2019.

ADVANCED SCIENCE
Open Access

Supporting Information

for *Adv. Sci.,* DOI: 10.1002/advs.201902371

Immobilization of Ni_3Co Nanoparticles into N-Doped Carbon Nanotube/Nanofiber Integrated Hierarchically Branched Architectures toward Efficient Overall Water Splitting

Tongfei Li, Sulin Li, Qianyu Liu, Jingwen Yin, Dongmei Sun, Mingyi Zhang, Lin Xu, Yawen Tang, and Yiwei Zhang**

Copyright WILEY-VCH Verlag GmbH & Co. KGaA, 69469 Weinheim, Germany, 2019.

Supporting Information

Immobilization of Ni_3Co nanoparticles into N-doped carbon nanotube/nanofiber integrated hierarchically branched architectures towards efficient overall water splitting

Tongfei Li, Sulin Li, Qianyu Liu, Jingwen Yin, Dongmei Sun, Mingyi Zhang, Lin Xu, Yawen Tang and Yiwei Zhang**

T. Li, S. Li, Q. Liu, J. Yin, Prof. D. Sun, Prof. L. Xu, Prof. Y. Tang
Jiangsu Key Laboratory of New Power Batteries, Jiangsu Collaborative Innovation Centre of Biomedical Functional Materials, School of Chemistry and Materials Science, Nanjing Normal University, Nanjing 210023, China
E-mail: xulin001@njnu.edu.cn, njuxulin@gmail.com (L. Xu)

T. Li, Prof. Y. Zhang
School of Chemistry and Chemical Engineering, Southeast University, Jiangsu Optoelectronic Functional Materials and Engineering Laboratory, Nanjing 211189, China
E-mail: zhangchem@seu.edu.cn (Y. Zhang)

Prof. M. Zhang
Key Laboratory for Photonic and Electronic Bandgap Materials, Ministry of Education, School of Physics and Electronic Engineering, Harbin Normal University, Harbin 150025, P. R. China

Experimental Section

Chemicals and reagents

Nickel (Ⅱ) nitrate hexahydrate ($Ni(NO_3)_2{\cdot}6H_2O$), Cobalt (Ⅱ) nitrate hexahydrate ($Co(NO_3)_2{\cdot}6H_2O$) and Polyvinyl pyrrolidone (PVP, *M*w ≈ 1 300 000) were obtained from Alfa Aesar. N, N-dimethylformamide (DMF), ethanol (C_2H_5OH) were purchased from Sinopharm Chemical Reagent Co., Ltd. All chemical reagents were used as received without further purification.

Synthesis of $Ni_{1.5}Co_{0.5}$@N-C NT/NFs

For the preparation of the standard $Ni_{1.5}Co_{0.5}$@N-C NT/NFs, 1.0 g of PVP was initially dissolved in 6 mL of DMF and 6 mL of ethanol to form transparent viscous solution. After that, 1.5 mmol of $Ni(NO_3)_2{\bullet}6H_2O$ and 0.5 mmol of $Co(NO_3)_2{\bullet}6H_2O$ were introduced into the above solution to form homogeneous chocolate sol after stirring for ~12 h. The resultant sol was then transferred into a plastic syringe equipped with a 21-gauge needle at the tip. The distance between the needle tip and fiber collector (aluminum foil) was set as 18 cm, and a high voltage of 20 kV was applied. The feeding rate of the sol was maintained at 0.6 mL h^{-1} with the assistance of a syringe pump. The as-spun fibers were subsequently stabilized in air at 200 ^oC for 3 h and further underwent calcination at 700 ^oC under a flow of 5% H_2 /95% Ar for 6 h, leading to the formation of $Ni_{1.5}Co_{0.5}$@N-C NT/NFs.

For comparison, a range of reference samples were also synthesized according to the similar protocol of the standard product, except adjusting the synthetic parameters, including pyrolysis temperature, feeding ratio and amount of metal precursors. To unravel the influence of pyrolysis temperature, the as as-spun fibers were also calcinated at 600, 650, and 750 ^oC, respectively, while other synthetic parameters were kept unchanged. To investigate the effect of feeding ratio of the metal precursors, only 2.0 mmol of $Ni(NO_3)_2$ led to the formation of Ni@N-C NT/NFs and only introduction of 2.0 mmol of $Co(NO_3)_2$ resulted in the generation of Co@N-CNFs. Metal precursors containing 0.5 mmol of Ni^{2+}/1.5 mmol of Co^{2+} and 1.0

mmol of Ni^{2+}/1.0 mmol of Co^{2+} generate $Ni_{0.5}Co_{1.5}$@N-CNFs and $Ni_{1.0}Co_{1.0}$@N-CNFs, respectively. Moreover, the influence of total amount of metal precursors was also examined. 1.0 mmol of Ni^{2+}/0.33 mmol of Co^{2+} and 2.0 mmol of Ni^{2+}/0.67 mmol of Co^{2+} brought about the formation of $Ni_{1.0}Co_{0.33}$@N-C NT/NFs and $Ni_{2.0}Co_{0.67}$@N-CNFs, respectively. N-doped carbon nanofibers (N-CNFs) were prepared by the direct pyrolysis of PVP nanofibers, without involving any metal precursors.

Materials Characterization

X-ray diffraction (XRD) patterns were performed on a Model D/max-rC X-ray diffractometer with Cu Kα radiation (λ = 1.5406 Å). Transmission electron microscopy (TEM) and high-resolution TEM (HRTEM) were taken on a JEOL JEM-2100F with an accelerating voltage of 200 kV. Field-emission scanning electron microscopy (FESEM) images were acquired on a JEOL JSM7500F. High-angle annular dark-field scanning TEM (HAADF-STEM) images, elemental mapping images and electron energy dispersive spectroscopy (EDS) were characterized on FEI Tecnai G2 F20 microscope, equipped with an accessory built on the JEOL JEM-2100F. Thermogravimetric analysis (TGA) was recorded on a NetzschSTA449C thermal analyzer with a ramp rate of 10 °C min^{-1} under O_2 atmosphere. X-ray photoelectron spectroscopy (XPS) was analyzed on a Thermo VG Scientific ESCALAB 250 spectrometer with an Al Kα radiator. Raman spectra were obtained on a Raman spectrometer (Lab RAM HR800, λ = 514 nm). N_2 sorption isotherms and Brunauer-Emmett-Teller (BET) surface areas were measured on Micromeritics ASAP 2050 instrument.

Electrochemical Measurements

The electrochemical measurements for HER and OER were performed on a CHI 760E workstation in a conventional three-electrode system. For the configuration of the three-electrode system, a catalyst-modified glassy carbon electrode (d = 3 mm), a saturated calomel electrode (SCE) and a graphite rod were used as the working electrode, the reference

electrode and the counter electrode, respectively. To prepare the catalyst ink, 4.0 mg of the obtained catalyst was ultrasonically dispersed in a mixed solvent containing 1.5 mL H_2O, 0.5 mL C_2H_5OH and 5 μL Nafion (5 wt%) solution for 30 min. Afterwards, 20 μL of the above suspension was dropped onto the glassy carbon electrode surface and then dried at room temperature, and the mass loading density of the active material in glassy carbon electrode was ~0.56 mg/cm^2. The linear-sweep voltammograms (LSV) were carried out at a scan rate of 5 mV s^{-1} in N_2-saturated electrolyte for HER and O_2-saturated electrolyte for OER in 0.1 M KOH solution. All potentials in this work were referenced to reversible hydrogen electrode (RHE), using the following equation: $E_{RHE} = E_{SCE} + 0.0592\ pH + 0.242$.

The evaluation of overall water splitting was carried out in a two-electrode system, and two pieces of nickel foams (3 cm ×1 cm) were employed to support the Ni_3Co@N-C NT/NF catalyst. Before the immobilization of catalyst, the nickel foams were thoroughly cleaned with acetone, 6.0 M HCl and copious amount of water. The pre-cleaned nickel foam piece was wrapped with Teflon tape at the 2 cm ×1 cm area from one end, leaving the rest 1 cm × 1 cm area exposed for the deposition of catalyst. The catalyst ink was prepared by dispersing 5 mg of the catalyst into 0.1 mL of ethanol, 0.1 mL of H_2O, and 50 μL of 5 wt% Nafion solution under sonication for 1 h. Subsequently, 100 μL of the catalyst ink was drop-cast onto the exposed area of the nickel foam piece and air-dried under ambient conditions. The loading mass of the catalyst on the nickel foam substrate is ~2 mg/cm^2. The catalyst-modified nickel foam pieces were assembled as the negative electrode for HER and the positive electrode for OER, respectively. The overall water splitting was performed in 1.0 M KOH electrolyte at a scan rate of 5 mV s^{-1}.

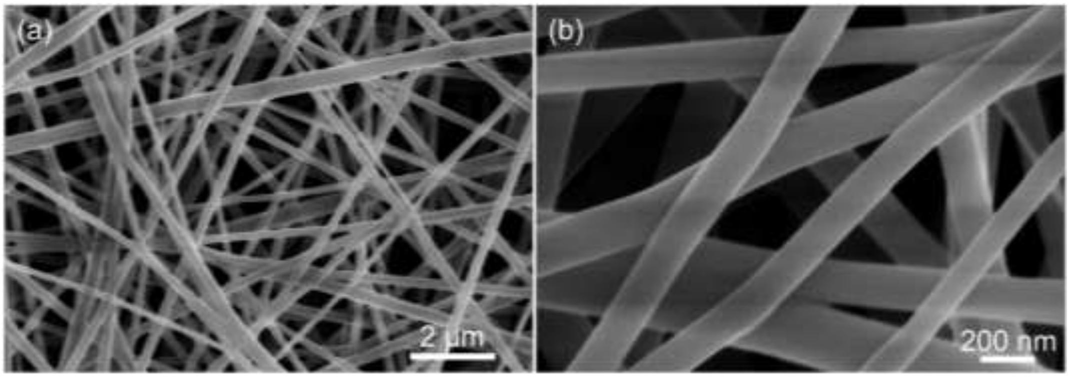

Figure S1. SEM images of the synthesized polymer nanofibers with different magnifications.

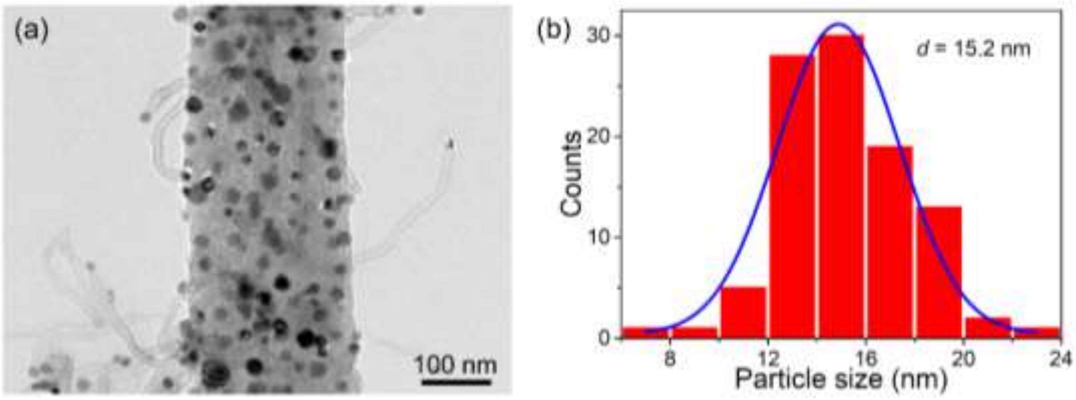

Figure S2. (a) Representative TEM image and (b) particle size distribution of the encapsulated Ni_3Co nanoparticles of the fabricated $Ni_{1.5}Co_{0.5}$@N-C NT/NFs.

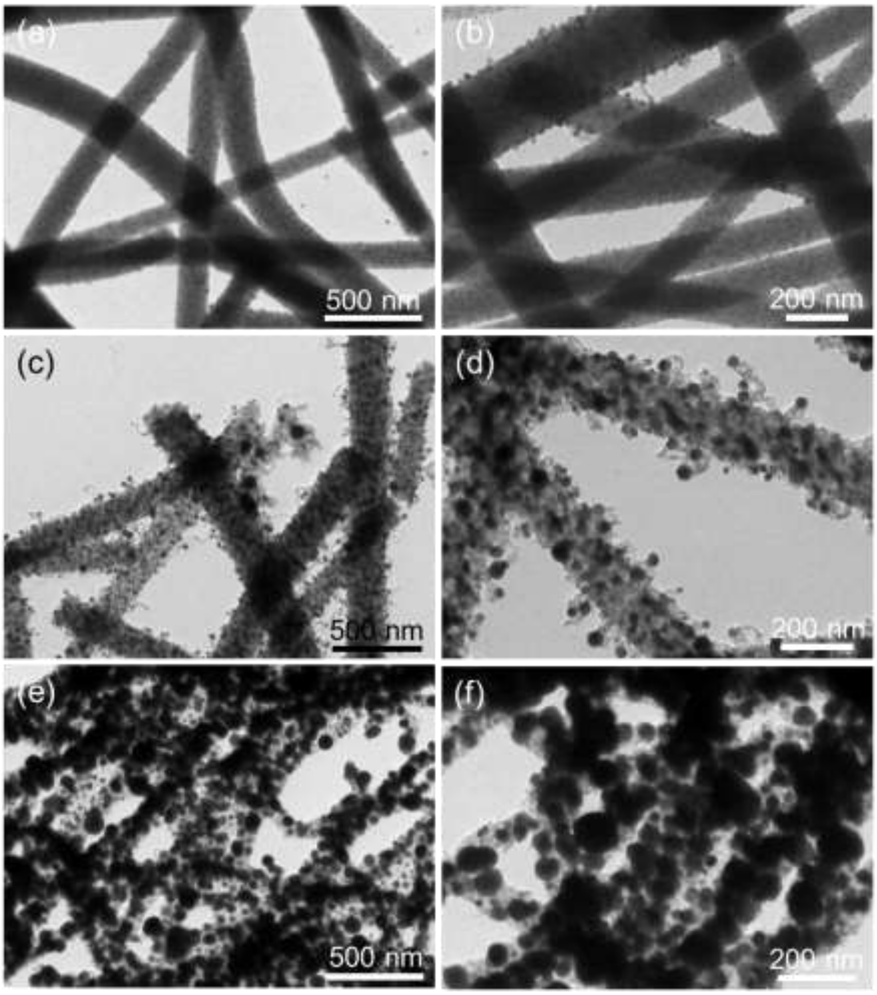

Figure S3. TEM images of the hybrid nanofibers obtained in the presence of different calcination temperature. (a)-(b) 600 ^{o}C, (c)-(d) 650 ^{o}C and (e)-(f) 750 ^{o}C.

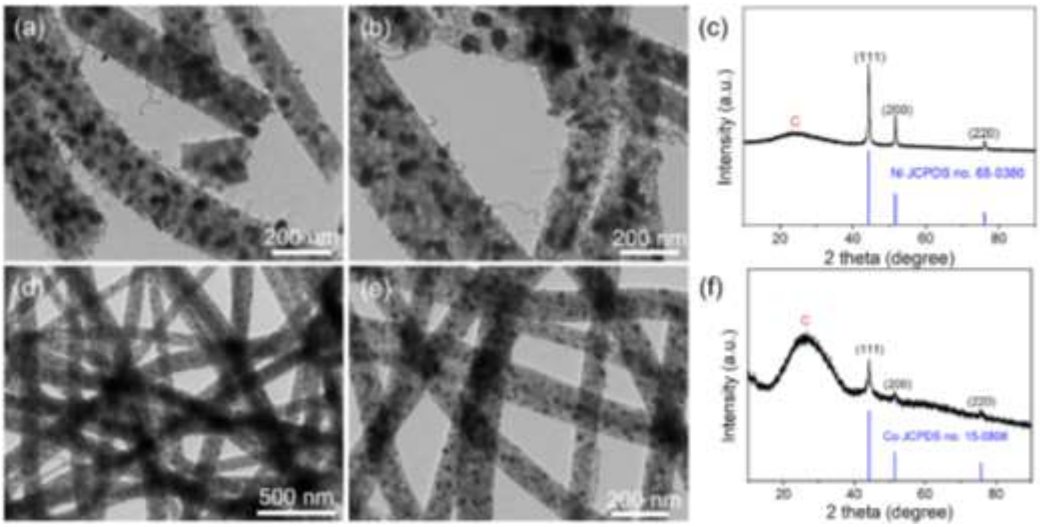

Figure S4. (a)-(c) TEM images and XRD pattern of the synthesized Ni@N-C NT/NFs, and (d)-(f) TEM images and XRD pattern of the obtained Co@N-CNFs.

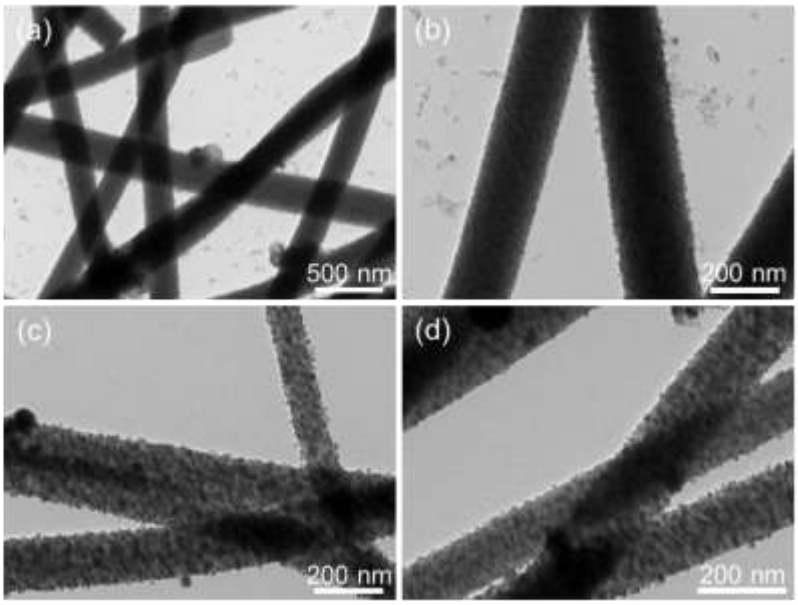

Figure S5. TEM images of the hybrid nanofibers obtained in the presence of different ratio of metal precursors. (a)-(b) 0.5 mmol of $Ni(NO_3)_2$ + 1.5 mmol of $Co(NO_3)_2$, (c)-(d) 1.0 mmol of $Ni(NO_3)_2$ + 1.0 mmol of $Co(NO_3)_2$.

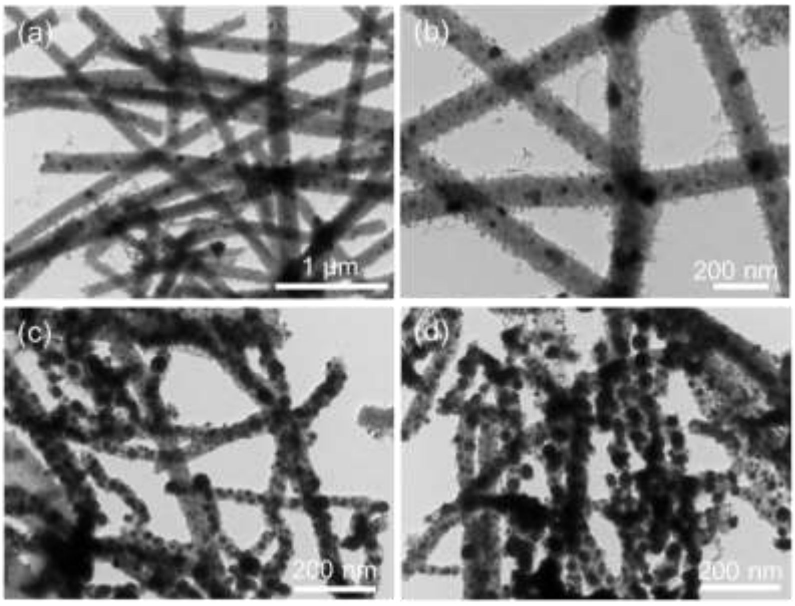

Figure S6. TEM images of the hybrid nanofibers obtained in the presence of different amount of metal precursors. (a)-(b) 1.0 mmol of $Ni(NO_3)_2$ + 0.33 mmol of $Co(NO_3)_2$ and (c)-(d) 2.0 mmol of $Ni(NO_3)_2$ + 0.67 mmol of $Co(NO_3)_2$.

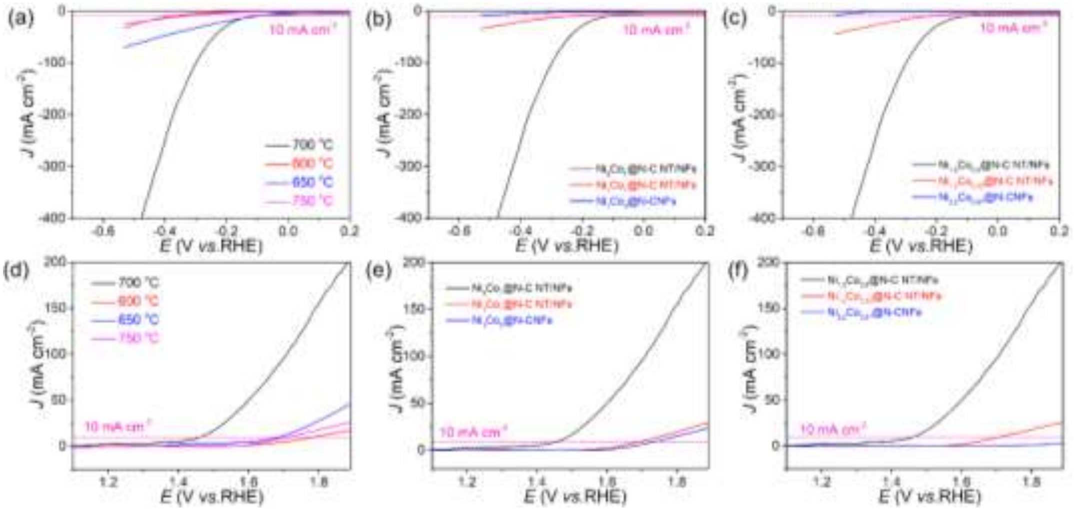

Figure S7. HER and OER LSV curves of the different synthesized catalysts synthesized at different synthetic conditions. (a, d) different calcination temperatures, (b, e) different molar ratios of the Ni and Co, and (c, f) different feeding amount of the Ni and Co.

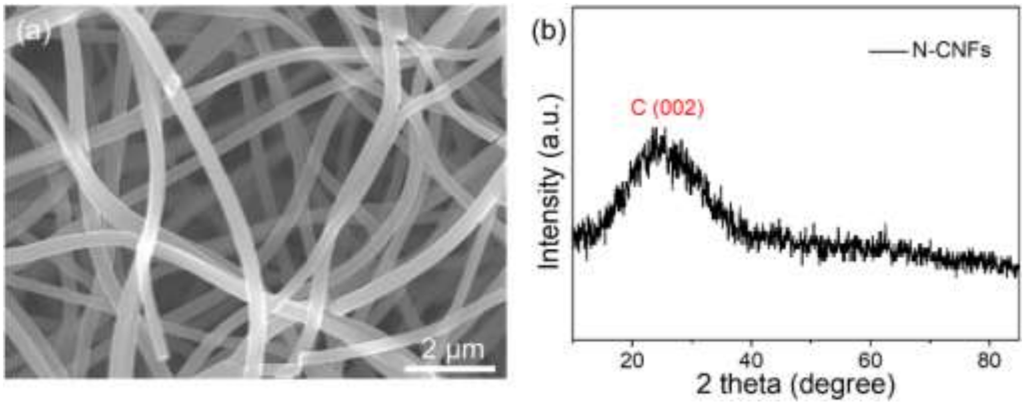

Figure S8. (a) SEM image and (b) XRD pattern of the resultant N-CNFs.

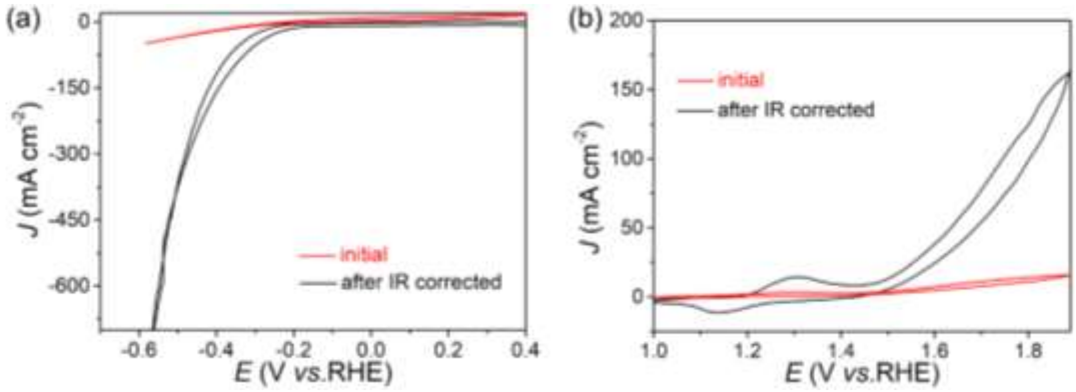

Figure S9. CVs recorded for GC electrodes modified with Ni_3Co@N-C NT/NFs with (black) and without (red) iR correction for (a) HER and (b) OER.

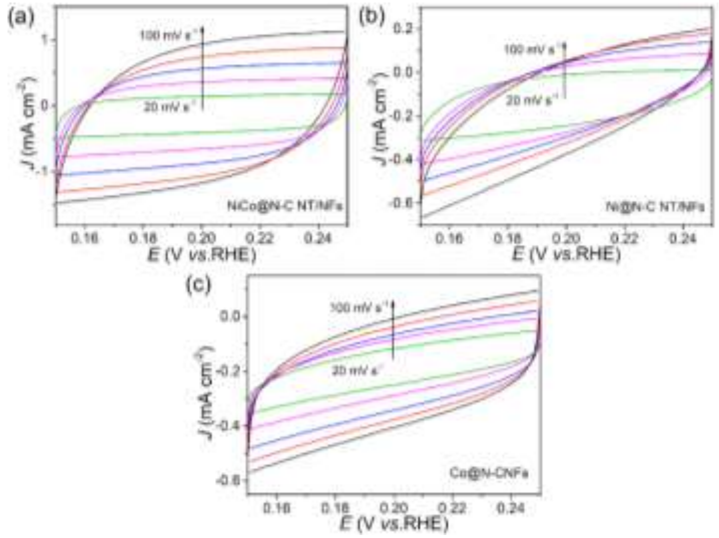

Figure S10. CV curves of the synthesized catalysts in the non-Faradaic region (0.15 - 0.25 V *vs* RHE) obtained at different scanning rates. (a) standard $Ni_{1.5}Co_{0.5}$@N-C NT/NFs, (b) Ni@N-C NT/NFs, (c) Co@N-CNFs.

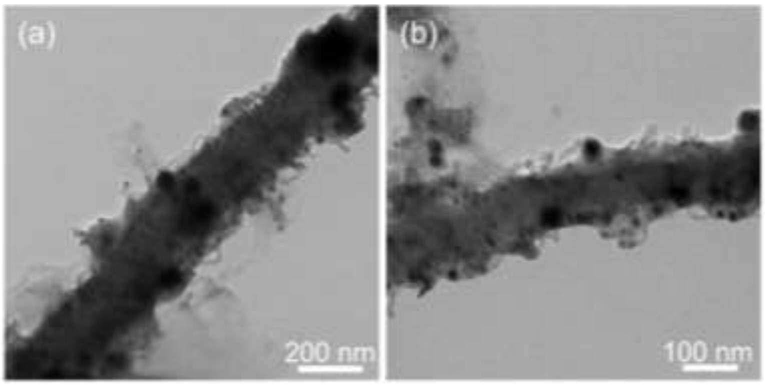

Figure S11. TEM images of the $Ni_{1.5}Co_{0.5}$@N-C NT/NF catalyst after HER stability test.

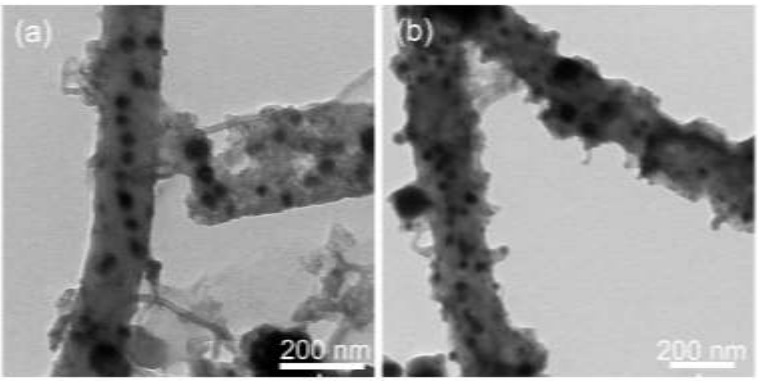

Figure S12. TEM images of the $Ni_{1.5}Co_{0.5}$@N-C NT/NF catalyst after OER stability test.

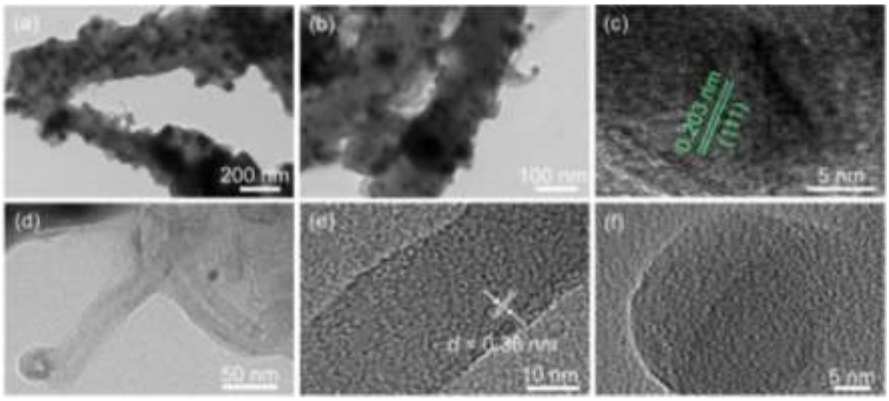

Figure S13. Structural characterization of the Ni_3Co@N-C NT/NFs after the long-term water electrolysis test. (a)-(b) TEM images, (c) HRTEM image of an individual Ni_3Co nanoparticle, and (d)-(f) HRTEM images of a single carbon nanotube.

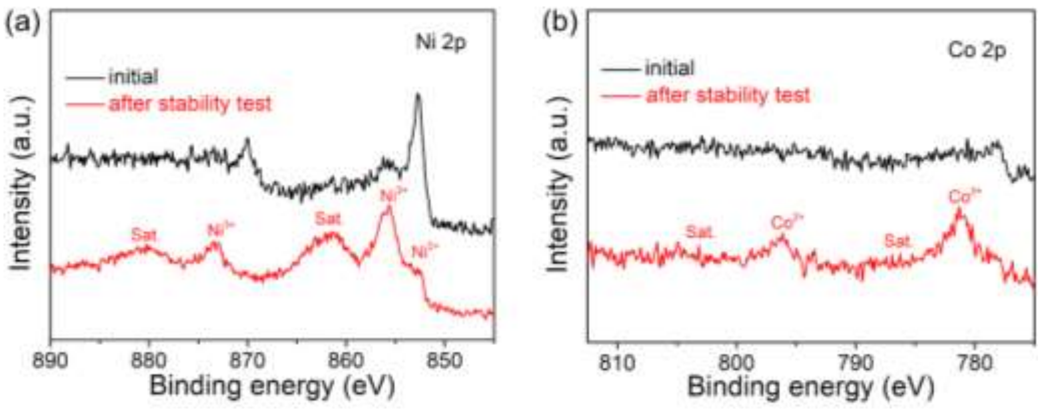

Figure S14. XPS spectra of Ni_3Co@N-C NT/NFs before and after stability test. (a) Ni 2p region and (b) Co 2p region.

Table S1. Comparison of HER performance of $Ni_{1.5}Co_{0.5}$@N-C NT/NFs with some previously reported non-noble catalysts in alkaline solution.

Catalysts	Overpotential @ 10 mA cm^{-2} (mV)	Tafel slope (mV dec^{-1})	Ref.
$Ni_{1.5}Co_{0.5}$@N-CNT/NFs	114	117	This work
CA-$Ni_{0.25}V_{0.75}$S/NF	150	101	*Appl. Catal. B: Environ.* **2019**, *259*, 117984.
CoMoOS	123	86	*Nanoscale* **2019**, *11*, 3500.
Ni_2P-Cu_3P@NiCu	118	185	*ACS Catal.* **2019**, *9*, 6919.
Co-Ni_3N	194	156	*Adv. Mater.* **2018**, *30*, 1705516.
NiS-Ni_9S_8- NiSe-NR/NF	112	94.1	*ACS Appl. Mater. Interfaces* **2018**, *10*, 2430.
Ni_3Se_4	203	156	*ACS Appl. Mater. Interfaces* **2017**, *9*, 8714.
NiFe LDH	210	N/A	*Angew. Chem. Int. Ed.* **2016**, *55*, 15240.
$Ni_{0.9}Fe_{0.1}$/NC-NF	231	111	*ACS Catal.* **2016**, *6*, 580.
Co_9S_8-MoS_2/CNFs	190	110	*Adv. Mater.* **2015**, *27*, 4752.
CoMnO@CN	71	152	*J. Am. Chem. Soc.* **2015**, *137*, 14305.
Ni_3S_2 Nanosheet Arrays/NF	223	N/A	*J. Am. Chem. Soc.* **2015**, *137*, 14023.
$Co_{0.85}$Se/NiFe-LDH/EG	260	160	*ACS Nano* **2015**, *41*, 6278.
Co-P/Co-PO_4	380	N/A	*Adv. Mater.* **2015**, *27*, 3175.
Co_9S_8@CNFs	>400	203	*Adv. Mater.* **2015**, *27*, 4752.
CoP nanowires	209	129	*J. Am. Chem. Soc.* **2014**, *136*, 7587.
Ni_3S_2/CNT	N/A	167	*Appl. Catal. B: Environ.* **2014**, *7*, 213.
FeP NAs/CC	220	475	*ACS Catal.* **2014**, *4*, 4065.
$Ni(OH)_2$/NF	250	N/A	*Science* **2014**, *345*, 1593.

Table S2. Comparison of OER performance of $Ni_{1.5}Co_{0.5}$@N-C NT/NFs with some previously reported non-noble catalysts in alkaline solution.

Catalysts	Overpotential @ 10 mA cm^{-2} (mV)	Tafel slope (mV dec^{-1})	Ref.
$Ni_{1.5}Co_{0.5}$@N-C NT/NFs	243	103	This work
Fe@BIF-91	350	71	*Adv. Sci.* **2019**, *6*, 1801920.
Co/MnO@GC-700	358	98	*Nanoscale* **2018**, *10*, 13702.
Cu_7Te_4 nanosheets	460	103	*Nano Energy* **2017**, *41*, 780.
NiCoP/C	330	96	*Angew. Chem. Int. Ed.* **2017**, *56*, 3897.
Ni_3Se_2 films	310	97.1	*Energy Environ. Sci.* **2016**, *9*, 1771
Ni@C-400 NSs	330	145	*J. Mater. Chem. A* **2016**, *4*, 7297.
TiN@Ni_3N	350	94	*J. Mater. Chem. A* **2016**, *4*, 5713.
NCNF-1000	570	274	*Adv. Mater.* **2016**, *28*, 3000.
N/Co-doped PCP//NRGO	430	292	*Adv. Funct. Mater.* **2015**, *25*, 872.
Ni_3S_2 Nanosheet Arrays@NF	339	131	*J. Am. Chem. Soc.* **2015**, *137*, 14023.
CoS_2/N-S GO	370	N/A	*ACS Catal.* **2015**, *5*, 3625.
NG-CNT	414.5	128	*Adv. Sci.* **2015**, *2*, 1400015.
NiCo-LDH	~420	113	*J. Power Sources* **2015**, *278*, 445.
Ni-Co Hydroxide	460	145	*Adv. Funct. Mater.* **2014**, *24*, 4698.

Table S3. Comparison of the electrochemical performance of $Ni_{1.5}Co_{0.5}$@N-C NT/NFs with some previously reported non-noble catalysts for overall water splitting in alkaline solution.

Catalysts	Overpotential @ 10 mA cm^{-2} (V)	Ref.
NiCo@N-C NT/NFs	1.57	This work
CVN	1.64	*Appl. Catal. B: Environ.* **2019**, *241*, 521.
Ni_3N@CoN	1.59	*J. Mater. Chem. A* **2018**, *6*, 4466.
NiMoP	1.60	*ACS Appl. Mater. Interfaces* **2018**, *10*, 29647.
$Ni_{0.75}Fe_{0.125}V_{0.125}$-LDHs/NF	1.591	*Small* **2018**, *14*, 1703257.
Ni–Co–P HNBs	1.62	*Energy Environ. Sci.* **2018**, *11*, 872.
PO-Ni/Ni-N-CNFs	1.69	*Nano Energy* **2018**, *51*, 286.
(Ni,Co)Se_2-GA	1.60	*ACS Catal.* **2017**, *7*, 6394.
NiMoN-550	1.596	*J. Mater. Chem. A* **2017**, *5*, 13648.
Co_4NiP NTs	1.59	*Adv. Funct. Mater.* **2017**, *27*, 1703455.
Ni@NC	1.60	*Adv. Mater.* **2017**, *29*, 1605957.
$Ni_{0.69}Co_{0.31}$-P	1.59	*Nanoscale* **2016**, *8*, 19129.
$NiCo_2O_4$ hollow microcuboids	1.65	*Angew. Chem. Int. Ed.* **2016**, *55*, 1.
$NiCo_2S_4$ NW/NF	1.63	*Adv. Funct. Mater.* **2016**, *26*, 4661.
Ni_3S_2 Nanosheet Arrays/NF	1.76	*J. Am. Chem. Soc.* **2015**, *137*, 14023
Ni_2P/NiOx	~1.63	*Energy Environ. Sci.* **2015**, *8*, 2347.
NiFe LDH/NF	1.7	*Science* **2014**, *345*, 1593.
